# Preclinical Assessment of a Novel Polymer-Free Hybrid Drug Eluting Stent

**DOI:** 10.1007/s12265-025-10680-5

**Published:** 2025-08-15

**Authors:** Léa Wild, Alicia Beele, Masaru Seguchi, Tobias Lenz, Philipp Nicol, Emina Sabic-Halilcevic, Grace R. Klosterman, Adnan Kastrati, Michael Joner

**Affiliations:** 1 Department of Cardiovascular Diseases, German Heart Center Munich, Technical University Munich University Hospital, Lazarettstraße 36, 80636 Munich, Germany; 2 DZHK (German Center for Cardiovascular Research), Partner Site Munich Heart Alliance, Berlin, Germany; 3 Division of Cardiovascular Medicine, Saitama Medical Center, Jichi Medical University, Saitama, Japan

**Keywords:** Drug-eluting stent, Percutaneous coronary intervention, Porcine model, Vascular healing

## Abstract

We aimed to evaluate a novel polymer-free hybrid drug eluting stent (DES) relative to benchmark devices and specific controls addressing singular components of the test device in a juvenile pig model of coronary stenting. 80 stents were implanted in 28 juvenile pigs and evaluated at 28 and 90 days using quantitative coronary angiography and histopathology (*n* = 10 per group). Scanning electron microscopy was used at 14 days to assess early re-endothelialization (*n* = 3 per group). The test device featured a cobalt-chromium (CoCr) backbone with a polymer-free probucol matrix releasing everolimus. The polymer-free test stent showed improved strut coverage at 28 days compared to the polymer-coated control, with significantly lower neointimal growth at 90 days and near complete endothelialization at 14 days. This preclinical study supports the favorable vascular healing profile of a polymer-free hybrid DES, warranting further clinical investigation.

**Graphical Abstract:**

Preclinical assessment of a novel polymer-free DES. The polymer-free coating strategy demonstrated a favorable healing profile in both short- and mid-term evaluation compared to polymer-based benchmark devices

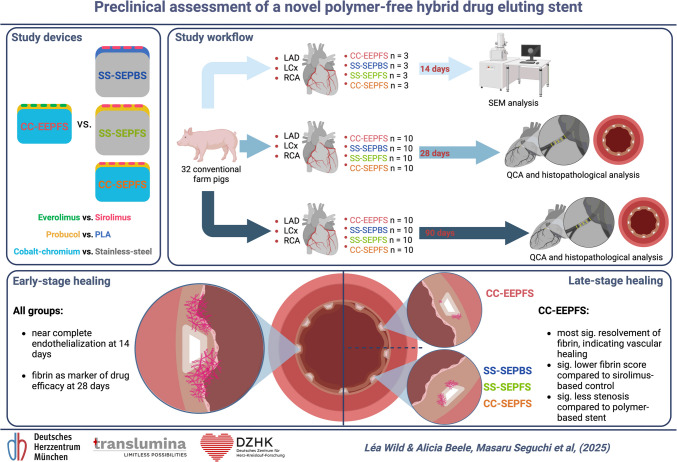

**Supplementary Information:**

The online version contains supplementary material available at 10.1007/s12265-025-10680-5.

## Introduction

The introduction of coronary stent implantation improved the durability of arterial patency following percutaneous coronary intervention (PCI) by enhancing acute gain, reducing acute vessel closure, plaque prolapse and arterial recoil, and inhibiting constrictive vascular remodelling [1]. The introduction of DES releasing anti-proliferative substances from the stent carrier matrix resulted in a substantial reduction of in-stent restenosis owing to excessive neointimal hyperplasia within the stented coronary segment of bare metal stents (BMS) [2]. Yet, DES implantation has also been associated with delayed arterial healing, characterized by delayed endothelialization, sustained inflammation and an increased risk of late thrombotic events relative to BMS [3]. Furthermore, the chronic delay in vascular healing was also believed to provide a nidus for the formation of in-stent neoatherosclerosis, which refers to the development of new atherosclerotic lesions within the stented neointimal tissue after DES implantation [4]. Hence, several attempts were undertaken to reduce the burden of foreign-body-related adverse vascular reactions resulting in the introduction of DES utilizing biodegradable polymer technology [5]. However, contemporary clinical trial results could not provide evidence for superiority of patients receiving DES with biodegradable polymers with regards to clinical and angiographic endpoints [6], whilst showing non-inferiority in this regard [7, 8]*.* Against this background, it was our goal to investigate a novel DES avoiding polymer coating technology to enable delayed drug release, and to compare against control DES utilizing biodegradable polymer coating. Furthermore, the present study aimed to compare this novel DES with two additional control devices to investigate whether the change of backbone, carrier matrix and drug impacts on vascular healing.

## Material and Methods

### Study Devices

**Fig. 1:**
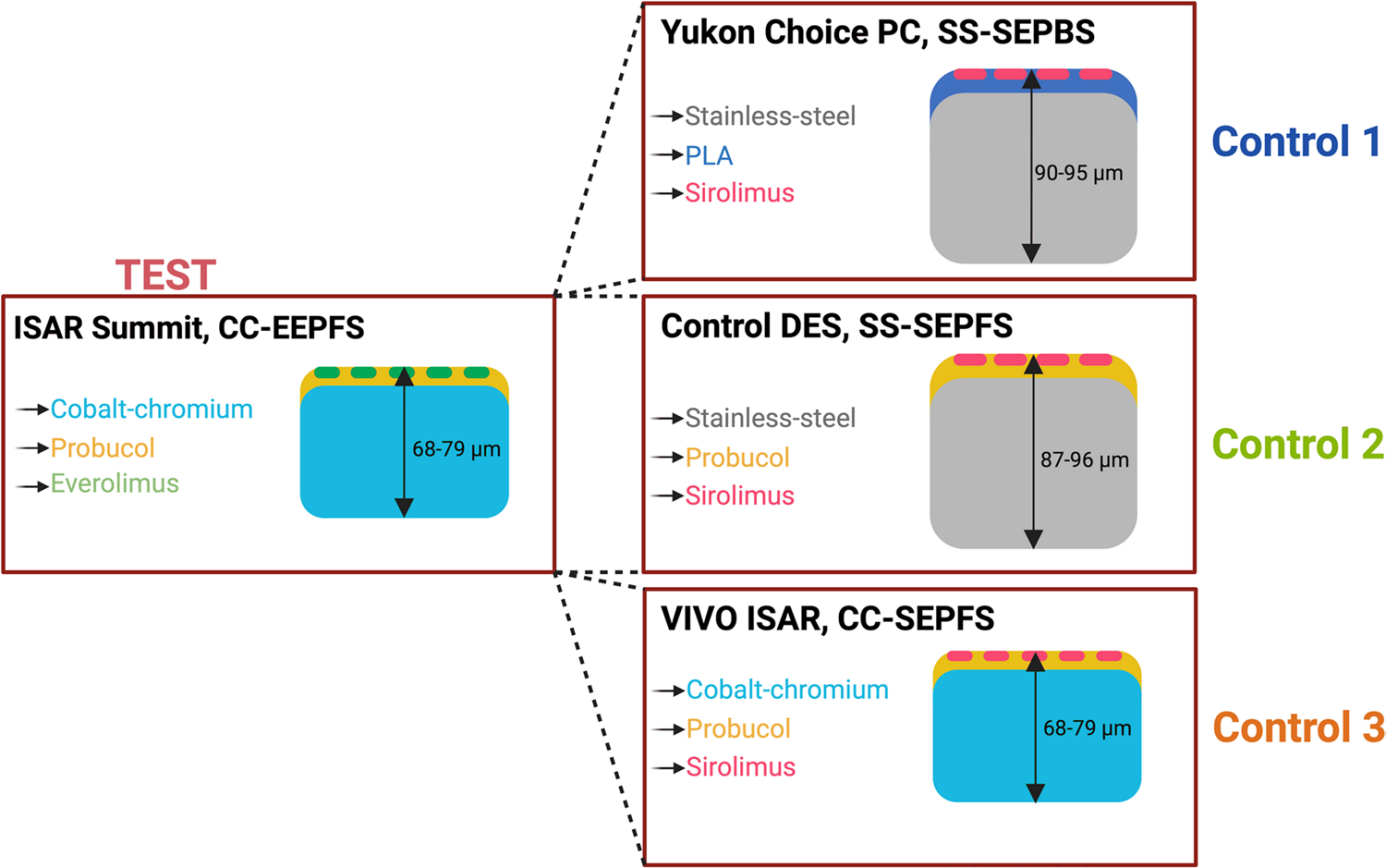
Overview of the different study devices. Devices differed in following main stent components: metallic backbone, drug carrier matrix and anti-proliferative drug

The basic characteristics of each device are visualized in Fig. 1. Additionally, more detailed information about e.g. drug load or strut thickness can be found in supplementary Table 1. Implanted stent sizes are depicted in supplementary Table 2.

CC-EEPFS (Cobalt Chromium Everolimus Eluting Polymer Free Stent, Test Device, ISAR Summit, *n* = 24) consisted of a novel thin-strut CoCr based everolimus-eluting stent (2.6 µg/mm^2^ everolimus) using probucol as excipient for delayed drug release.

SS-SEPBS (Stainless Steel Sirolimus Eluting Polymer Based Stent, Control 1, Yukon® Choice PC, biodegradable polymer-based DES *n* = 24, commercially available), a well-established older generation DES, differed in all three major components and consisted of a SS sirolimus-eluting stent (2.6 µg/mm^2^ sirolimus) using biodegradable polymer (PLA, polylactic acid) as matrix for delayed drug release.

SS-SEPFS (Stainless Steel Sirolimus Eluting Polymer Free Stent, Control 2, *n* = 23, designed specifically for this study and not commercially available) differed in two components and consisted of a SS sirolimus-eluting stent (2.6 µg/mm^2^ sirolimus) using probucol as excipient for delayed drug release.

CC-SEPFS (Cobalt Chromium Sirolimus Eluting Polymer Free Stent, Control 3, Vivo ISAR, *n* = 24, commercially available) differed in only one component and consisted of a CoCr backbone and probucol matrix with sirolimus (2.6 µg/mm^2^ sirolimus).

The strut thickness was 68 µm (devices for small vessels) or 79 µm (devices for medium vessels) for the CoCr-groups (CC-EEPFS and CC-SEPFS) and 90 µm/87 µm (devices for small vessels) or 95 µm/96 µm (devices for medium vessels) for SS-SEPBS/SS-SEPFS (SS-groups).

### Study Design

**Fig. 2:**
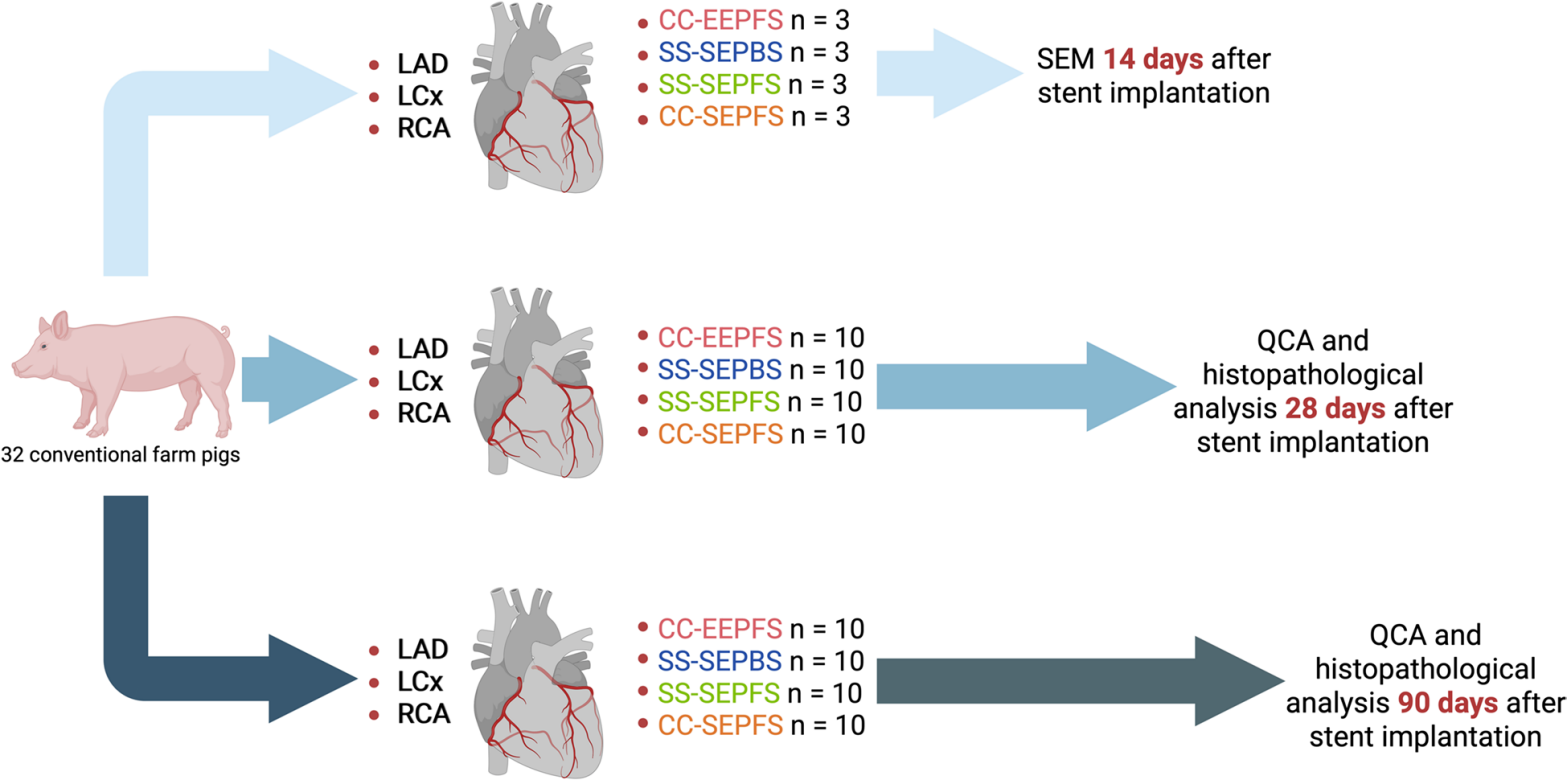
Schematic overview of the study design. 92 stents were successfully implanted in the coronary arteries of 32 conventional farm pigs. SEM analysis of *n* = 3 stents per group was performed 14 days after stent implantation. QCA and histopathological analysis was performed at short- (28 days) and mid-term (90 days) time points. LAD = left anterior descending, LCx = left circumflex, RCA = right coronary artery, SEM = scanning electron microscopy, QCA = quantitative coronary angiography

Ten stents from each of the four different DES types were randomly implanted in the coronary arteries of 28 healthy, juvenile domestic farm pigs for histopathological analysis at 28- and 90- days follow-up. Additionally, three stents from each group were randomly implanted in the coronary arteries of four healthy, juvenile domestic farm pigs for scanning electron microscopy at 14-days follow-up (see Fig. 2). Study pathologists were blinded until raw data collection was finished.

### Porcine Model of Coronary Stent Implantation

A total of 33 juvenile domestic farm pigs (castrated males, about 3 months old) were included in the study. Animals were received from Sauenhaltung Tierbach (Uthmöden, Germany) and were acclimatized for 14 days prior to use in the study. All animal experiments were performed in accordance with the requirements and guidelines of the directive 2010/63/EU and the German Animal Protection Act (in its current version).

### Interventional Procedures and Tissue Harvest

A loading dose of clopidogrel (75 mg) and aspirin (100 mg) was administered orally two days before treatment and long-acting Verapamil hydrochloride was given within 24 h prior to the procedure to prevent vascular spasm during catheterization.

After premedication with ketamine (20 mg/kg, i.m.) and xylazinhydrochloride 2% (2 mg/kg i.m.), pigs were put under general anesthesia using propofol 1% (3 mg/kg, i.v.). All animals received butorphanol (0.1 mg/kg, i.v.), meloxicam (0.4 ml/10 kg, i.m.) before intubation and ursocyclin as prophylactic antibiosis (1 ml/5 kg). Maintenance of anesthesia was performed by ventilation with a mixture of 30–60 vol% of pure oxygen, 40–70 vol% air and 1–2 vol% of Isoflurane. After sheath insertion in the carotid artery, heparin-natrium (5000 IU) and D,L-Lysinacetylsalicylat (250 mg) were administered intraarterially. Additional heparin doses were given as needed.

Angiographic imaging was performed before and after stent implantation, after application of glyceroltrinitrat (200 µg, i.c.). At day 0, the randomly assigned devices were implanted in the left anterior descending (LAD), left circumflex (LCx) and right coronary artery (RCA) of each animal with an overstretch of approximately 10% relative to the reference diameter of the vessel (overstretch ratio of 1.1:1). After stent implantation, coronary angiography was performed to document vessel patency and absence of residual dissection. Animals were sacrificed at 14-, 28- and 90-days following stent implantation after final angiography and complete necropsy was performed. Immediately after explantation, the hearts as well as each coronary artery were flushed with isotonic NaCl and fixed with 10% buffered formalin (pressure fixation; infusion of the fixative at a constant fluid pressure (~ 100 mm Hg) in each coronary artery). After fixation, the coronaries were explanted with a short segment of native (non-stented) vessel at the proximal and distal end. Vessel segments were marked with a suture tag at the proximal end of the vessel and photographed before processing.

### Quantitative Coronary Angiography (QCA)

Angiographic images were acquired with contrast media to identify an appropriate location for the stent implantation site. Computer-assisted software for offline analysis (Qangio XA7.3, Medis Medical Imaging Systems, Leiden, Netherlands) was used for angiographic analysis. The following parameters were measured or calculated: percentage diameter stenosis, mean lumen diameter, minimum lumen diameter. Late lumen loss was calculated as the difference in minimum lumen diameter between post-procedure and follow-up.

### Histopathological Evaluation

Vessels were embedded in methyl methacrylate (MMA) resin and divided into proximal (non-stented), proximal middle, middle, distal middle and distal (non-stented) blocks. Tissue blocks were then cut at 10 μm using a laser microtome (TissueSurgeon, LLS ROWIAK LaserLabSolutions GmbH, Hannover, Germany). Sections were stained with hematoxylin and eosin (H&E) and Verhoeff van Gieson (VVG). Histology slides were photographed and digitalized using an Olympus microscope (BX 41, Olympus, Tokyo, Japan) with associated camera (DP 74, Olympus, Tokyo, Japan). Morphometric measurements were taken in VVG images using cellSens Imaging Software (cellSens Standard 1.17, Olympus, Tokyo, Japan). All sections were examined by light microscopy. Vascular injury following stent implantation was graded as described previously [9].

The different scores are summarized in supplementary tables 3, 4, 5. Fibrin deposition (identified as intense, homogenous pink stain) and inflammatory response were assessed as previously described [3]. Cross sectional measurements (Lumen area and internal elastic lamina (IEL) area) were performed in stented cross sections and used to calculate percentage stenosis with following formula: percentage stenosis = [1- (Lumen Area/IEL Area)] *100.

### Scanning Electron Microscopy (SEM)

Specimens were fixed in 10% buffered formalin for 48 h, followed by fixation in glycerol. Subsequently, they were bisected longitudinally, post-fixed in 100% glycerol, and gently rinsed with 0.1 M sodium phosphate buffer solution (pH 7.4). Afterwards, the samples were dehydrated in a graded series of ethanol and critical point dried. The luminal surface was exposed *en face* and sputter-coated with gold. Specimens were visualized using a Zeiss EVO MA 15 scanning electron microscope. Stent areas and uncovered areas were measured using ImageJ 1.53 k (National Institutes of Health, USA). Measurements were performed on consecutive stent strut rows identified on bisected stent halves. The area of discernable stent strut rows was measured using the polygon selection tool. The expected number of strut rows for each stent was provided by the manufacturer; rows where stent struts were buried under a dense layer of tissue were confirmed to exhibit complete endothelialization on high-power (200x) magnification and then classified as completely covered, and those with visible processing artifacts were excluded from analysis. Visible connector areas between struts were measured separately. Uncovered areas were measured accordingly. The percentage of uncovered stent strut area was first calculated per strut row and connector, and then summed up to represent the entire bisected stent half.

### Statistical Methods

For QCA analysis, mean and standard deviation (SD), as well as the median and first- and third quartile were determined for each parameter. Continuous variables with normal distribution were compared using an unpaired student t-test, while variables showing non-parametric distribution were compared using Wilcoxon Rank-Sum test (Mann–Whitney u test).

For histopathological assessment, continuous data are presented as median (25th – 75th percentiles). Categorical data are presented as absolute and relative frequencies. Hypothesis testing of differences between the groups was performed using the Wilcoxon rank-sum test for continuous variables and the Pearson χ2 test (or Fisher’s exact test where any expected cell count of the contingency table was < 5) for categorical variables. To account for the clustered nature of the data, a linear mixed model was used for the analysis of histology data. The model contained a fixed-effects term for the variable of interest and a random intercept as random-effects term for animals in case of cross-sectional analysis and as nested random-effects term for animals and cross-section for strut-level analysis.

A *p*-value less than 0.05 was considered statistically significant. Statistical analysis was performed using the R 4.1.0 Statistical Package (The R Foundation for Statistical Computing, Vienna, Austria).

## Results

At day 0, a total of 95 stents were implanted into the three major coronary arteries of 33 pigs. One animal died prematurely in the night following stent implantation. This left a total of 92 implants in 32 pigs available for SEM analysis at 14- and histopathological analysis at 28- and 90-days follow-up. For SEM analysis, *n* = 3 devices were allocated to each of the test and control groups. Regarding histopathological assessment, *n* = 10 devices were allocated per group at both timepoints.

### Quantitative Coronary Angiography

There was no significant difference between the test and control groups at 28 days follow-up regarding the percentage of diameter stenosis, late lumen loss, minimum lumen diameter and mean lumen diameter (see supplementary Table 6). At 90 days, CC-EEPFS showed a trend towards lower late lumen loss compared to SS-SEPBS (see supplementary Table 7, *p* = 0.06).

### Morphometry

Morphometric measurements performed at 28 days follow-up showed significantly higher neointimal thickness above struts in arteries implanted with CC-EEPFS (0.13 [0.09, 0.19] mm) compared to arteries implanted with SS-SEPBS (0.09 [0.07, 0.13] mm, *p* = 0.01). The percentage of uncovered struts was numerically lower in CC-EEPFS and CC-SEPFS (polymer-free DES) compared to SS-SEPBS and SS-SEPFS, yet without reaching statistical significance (see Table 1).

**Table 1:** Morphometric comparison of stented cross sections at 28 days follow-up. Analysis includes mean ± SD, median and interquartile range (IQR) of all stented cross sections

		CC-EEPFS (*n* = 10)	SS-SEPBS (*n* = 10)	SS-SEPFS (*n* = 10)	CC-SEPFS (*n* = 10)	*p* value CC-EEPFS vs. SS-SEPBS	*p* value CC-EEPFS vs SS-SEPFS	*p* value CC-EEPFS vs. CC-SEPFS
**Area EEL (mm**^**2**^**)**	**Mean ± SD**	6.80 ± 1.77	7.32 ± 1.62	6.37 ± 1.82	6.37 ± 1.82	0.09	0.84	0.29
	**Median (Q**_**25**_**, Q**_**75**_**)**	6.90 (5.34, 8.35)	6.68 (5.96, 8.93)	7.03 (4.77, 7.96)	7.03 (4.77, 7.96)			
**Area IEL (mm**^**2**^**)**	**Mean ± SD**	5.78 ± 1.56	6.32 ± 1.49	5.44 ± 1.58	5.44 ± 1.58	0.06	0.68	0.32
	**Median (Q**_**25**_**, Q**_**75**_**)**	5.70 (4.47, 7.20)	5.68 (5.27, 7.63)	5.96 (4.12, 6.74)	5.96 (4.12, 6.74)			
**Lumen area (mm**^**2**^**)**	**Mean ± SD**	4.77 ± 1.51	5.27 ± 1.38	4.43 ± 1.53	4.43 ± 1.53	0.07	0.49	0.30
	**Median (Q**_**25**_**, Q**_**75**_**)**	4.57 (3.57, 6.00)	4.82 (4.29, 6.49)	4.64 (3.20, 5.74)	4.64 (3.20, 5.74)			
**Area media (mm**^**2**^**)**	**Mean ± SD**	1.02 ± 0.38	1.00 ± 0.27	0.93 ± 0.32	0.93 ± 0.32	0.69	0.43	0.26
	**Median (Q**_**25**_**, Q**_**75**_**)**	0.99 (0.74, 1.22)	0.95 (0.83, 1.09)	0.94 (0.64, 1.13)	0.94 (0.64, 1.13)			
**Neointimal area (mm**^**2**^**)**	**Mean ± SD**	1.01 ± 0.38	1.05 ± 0.44	1.01 ± 0.39	1.01 ± 0.39	0.60	0.26	0.95
	**Median (Q**_**25**_**, Q**_**75**_**)**	0.91 (0.74, 1.21)	0.98 (0.77, 1.23)	0.93 (0.73, 1.15)	0.93 (0.73, 1.15)			
**% Stenosis overall**	**Mean ± SD**	18.14 ± 7.04	16.97 ± 6.65	20.14 ± 10.75	20.14 ± 10.75	0.42	0.18	0.23
	**Median (Q**_**25**_**, Q**_**75**_**)**	16.39 (13.94, 20.59)	15.80 (13.04, 18.99)	16.52 (14.05, 22.53)	16.52 (14.05, 22.53)			
**Thickness above struts (mm)**	**Mean ± SD**	0.16 ± 0.12	0.12 ± 0.10	0.17 ± 0.13	0.17 ± 0.13	0.01	0.09	0.51
	**Median (Q**_**25**_**, Q**_**75**_**)**	0.13 (0.09, 0.19)	0.09 (0.07, 0.13)	0.13 (0.08, 0.21)	0.13 (0.08, 0.21)			
**Thickness** **between struts** **(mm)**	**Mean ± SD**	0.12 ± 0.09	0.11 ± 0.30	0.14 ± 0.12	0.14 ± 0.12	0.16	0.17	0.27
	**Median (Q**_**25**_**, Q**_**75**_**)**	0.10 (0.06, 0.16)	0.10 (0.06, 0.15)	0.11 (0.05, 0.17)	0.11 (0.05, 0.17)			
**% Uncovered struts**	**Mean ± SD**	0.00 ± 0.00	0.56 ± 3.04	0.00 ± 0.00	0.00 ± 0.00	0.32	0.11	-
	**Median (Q**_**25**_**, Q**_**75**_**)**	0.00 (0.00, 0.00)	0.00 (0.00, 0.00)	0.00 (0.00, 0.00)	0.00 (0.00, 0.00)			

**Fig. 3:**
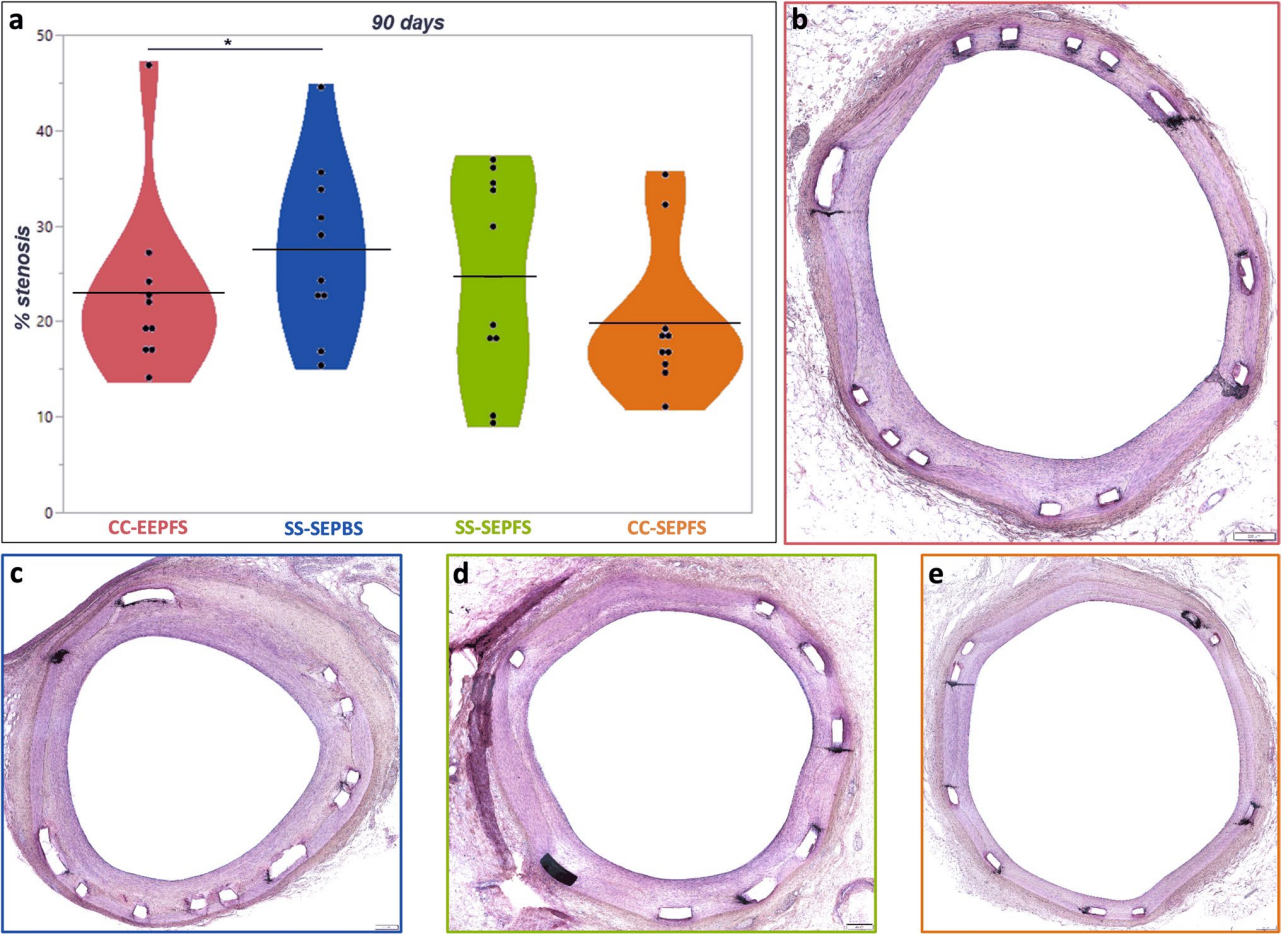
Morphometric analysis of percentage stenosis at 90 days follow-up. Percentage stenosis visualized in a violin plot with black bars indicating the mean value for each stent type. CC-EEPFS showed significant less percentage stenosis compared to SS-SEPBS (*p* = 0.03) (**a**). Representative low power images show less stenosis for CC-EEPFS (**b**) and CC-SEPFS (**e**) compared to SS-SEPBS (**c**) and SS-SEPFS (**d**). All images were taken at 4 × magnification. Scale bars depict 200 µm

At 90 days, CC-EEPFS showed significantly lower neointimal area (1.38 [0.89, 1.51] mm^2^) and overall percentage stenosis (19.55 [16.04, 25.50] %) compared to SS-SEPBS (1.63 [1.35, 1.91] mm^2^ and 26.37 [18.95, 31.39]%, with p < 0.001 and *p* = 0.03, respectively), while both polymer-free control devices (SS-SEPFS and CC-SEPFS) showed similar degree of neointimal growth (see Fig. 3). Neointimal thickness above and between struts was also significantly lower in CC-EEPFS (above: 0.16 [0.10, 0.24] mm, between: 0.14 [0.09, 0.21] mm; *p* = 0.001 and *p* = 0.01, respectively) compared to SS-SEPBS (above: 0.21 [0.15, 0.28] mm, between: 0.19 [0.13, 0.27] mm) (see Table 2).

**Table 2:** Morphometric comparison of stented cross sections at 90 days follow-up. Analysis includes mean ± SD, median and IQR of all stented cross sections

		CC-EEPFS (*n* = 10)	SS-SEPBS (*n* = 10)	SS-SEPFS (*n* = 10)	CC-SEPFS (*n* = 10)	*p* value CC-EEPFS vs. SS-SEPBS	*p* value CC-EEPFS vs SS-SEPFS	*p* value CC-EEPFS vs. CC-SEPFS
**Area EEL (mm**^**2**^**)**	**Mean ± SD**	7.35 ± 2.34	7.63 ± 2.11	6.94 ± 2.38	7.44 ± 1.74	0.63	0.79	0.87
	**Median (Q**_**25**_**, Q**_**75**_**)**	6.98 (5.01, 9.58)	7.76 (6.09, 8.25)	6.23 (5.36, 6.77)	7.72 (6.11, 8.88)			
**Area IEL (mm**^**2**^**)**	**Mean ± SD**	6.30 ± 2.08	6.62 ± 1.88	5.88 ± 2.03	6.44 ± 1.55	0.53	0.74	0.76
	**Median (Q**_**25**_**, Q**_**75**_**)**	6.02 (4.30, 8.18)	6.71 (4.94, 7.22)	5.22 (4.65, 8.82)	6.75 (5.30, 7.81)			
**Lumen area (mm**^**2**^**)**	**Mean ± SD**	4.99 ± 1.90	4.94 ± 1.91	4.59 ± 2.25	5.23 ± 1.54	0.72	0.72	0.60
	**Median (Q**_**25**_**, Q**_**75**_**)**	5.17 (3.52, 6.82)	5.01 (3.29, 5.58)	3.91 (3.22, 4.72)	5.74 (3.82, 6.43)			
**Area media (mm**^**2**^**)**	**Mean ± SD**	1.05 ± 0.32	1.01 ± 0.37	1.06 ± 0.42	0.99 ± 0.26	0.61	0.91	0.46
	**Median (Q**_**25**_**, Q**_**75**_**)**	1.10 (0.81, 1.24)	1.00 (0.75, 1.18)	1.00 (0.76, 1.28)	0.96 (0.84, 1.20)			
**Neointimal area (mm**^**2**^**)**	**Mean ± SD**	1.31 ± 0.47	1.68 ± 0.41	1.29 ± 0.53	1.22 ± 0.46	0.0007	0.88	0.41
	**Median (Q**_**25**_**, Q**_**75**_**)**	1.38 (0.89, 1.51)	1.63 (1.35, 1.91)	1.14 (0.93, 1.70)	1.18 (0.90, 1.32)			
**% Stenosis overall**	**Mean ± SD**	22.11 ± 9.20	27.23 ± 10.12	24.64 ± 13.23	19.82 ± 8.99	0.03	0.41	0.32
	**Median (Q**_**25**_**, Q**_**75**_**)**	19.55 (16.04, 25.50)	26.37 (18.95, 31.39)	21.63 (12.61, 33.05)	15.95 (14.48, 23.96)			
**Thickness above struts (mm)**	**Mean ± SD**	0.17 ± 0.10	0.23 ± 0.12	0.17 ± 0.13	0.18 ± 0.10	0.001	0.85	0.99
	**Median (Q**_**25**_**, Q**_**75**_**)**	0.16 (0.10, 0.24)	0.21 (0.15, 0.28)	0.13 (0.07, 0.26)	0.15 (0.09, 0.23)			
**Thickness** **between struts** **(mm)**	**Mean ± SD**	0.16 ± 0.09	0.21 ± 0.11	0.17 ± 0.12	0.16 ± 0.10	0.01	0.91	0.49
	**Median (Q**_**25**_**, Q**_**75**_**)**	0.14 (0.09, 0.22)	0.19 (0.13, 0.27)	0.13 (0.08, 0.24)	0.13 (0.08, 0.21)			
**% Uncovered struts**	**Mean ± SD**	0.00 ± 0.00	0.00 ± 0.00	0.00 ± 0.00	0.00 ± 0.00	-	-	-
	**Median (Q**_**25**_**, Q**_**75**_**)**	0.00 (0.00, 0.00)	0.00 (0.00, 0.00)	0.00 (0.00, 0.00)	0.00 (0.00, 0.00)			

### Histopathologic Response

**Fig. 4:**
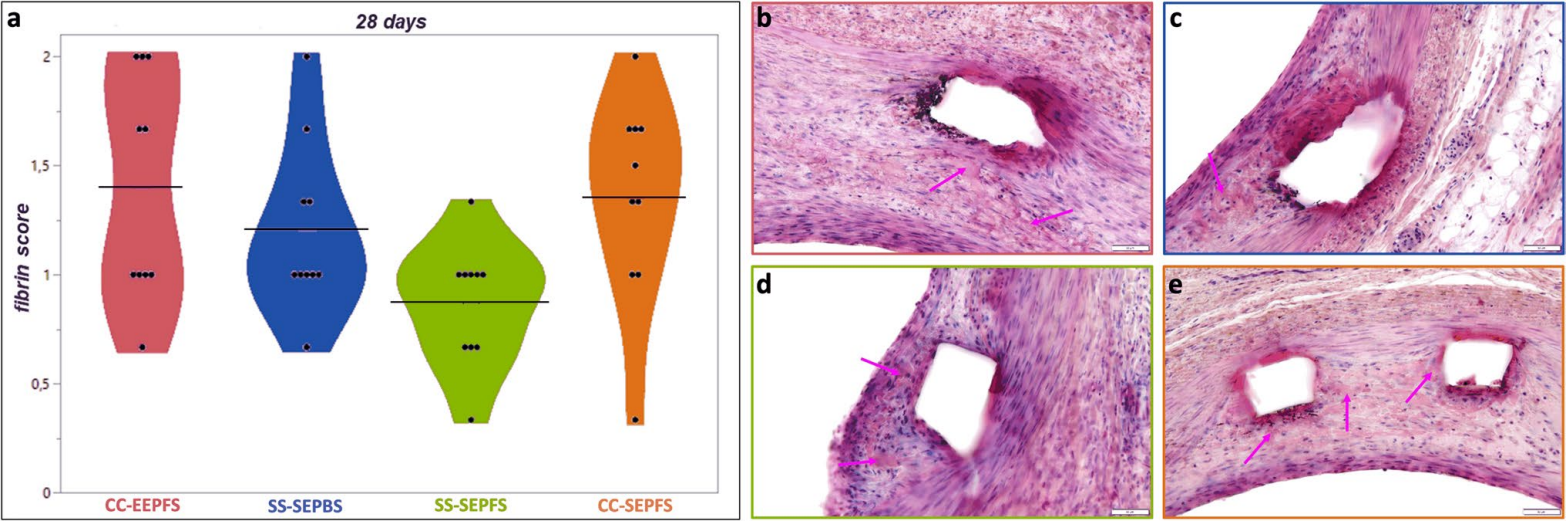
Histopathological analysis of fibrin score at 28 days follow-up. Fibrin score is visualized as a violin plot with the black bars indicating the mean value for each stent type. CC-EEPFS showed the highest fibrin score at 28 days, without reaching statistical significance (**a**). Representative high power images show mild to moderate fibrin deposition (pink arrows) adjacent to the stent struts in all four stent types (**b**-**e**). All images were taken at 20 × magnification. Scale bars depict 50 µm

At 28 days follow-up, fibrin score was highest in CC-EEPFS compared to all control devices, without reaching statistical significance (see Fig. 4). Arteries implanted with CC-EEPFS showed a higher percentage of struts with fibrin (85.16 [73.02, 100.00] %) compared to SS-SEPFS (50.00 [34.38, 69.17] %, *p* < 0.001) (see Table 3).

**Table 3:** Histologic comparison of vessel injury, healing and inflammatory response in stented cross sections at 28 days follow-up. Analysis includes mean ± SD, median and IQR of all stented cross sections

		CC-EEPFS (*n* = 10)	SS-SEPBS (*n* = 10)	SS-SEPFS (*n* = 10)	CC-SEPFS (*n* = 10)	*p* value CC-EEPFS vs. SS-SEPBS	*p* value CC-EEPFS vs. SS-SEPFS	*p* value CC-EEPFS vs.CC-SEPFS
**Injury Score**	**Mean ± SD**	1.16 ± 0.48	1.07 ± 0.30	1.02 ± 0.26	1.15 ± 0.45	0.09	0.22	0.89
	**Median (Q**_**25**_**, Q**_**75**_**)**	1.00 (1.00, 1.00)	1.00 (1.00, 1.00)	1.00 (1.00, 1.00)	1.00 (1.00, 1.00)			
**Injury Score circumferential Extension**	**Mean ± SD**	1.20 ± 0.48	1.13 ± 0.43	1.03 ± 0.18	1.21 ± 0.50	0.50	0.25	0.93
	**Median (Q**_**25**_**, Q**_**75**_**)**	1.00 (1.00, 1.00)	1.00 (1.00, 1.00)	1.00 (1.00, 1.00)	1.00 (1.00, 1.00)			
**Fibrin Score**	**Mean ± SD**	1.40 ± 0.56	1.20 ± 0.48	0.87 ± 0.43	1.32 ± 0.67	0.08	0.06	0.61
	**Median** **(Q**_**25**_**, Q**_**75**_**)**	1.00 (1.00, 2.00)	1.00 (1.00, 1.00)	1.00 (1.00,1.00)	1.00 (1.00, 2.00)			
**% of Struts with Fibrin**	**Mean ± SD**	83.38 ± 16.47	80.11 ± 15.86	50.93 ± 20.57	84.10 ± 20.55	0.32	0.0009	0.85
	**Median (Q**_**25**_**, Q**_**75**_**)**	85.16 (73.02, 100.00)	82.58 (75.00, 88.54)	50.00 (34.38, 69.17)	89.44 (83.46, 100.00)			
**Inflammation score**	**Mean ± SD**	1.30 ± 0.92	1.10 ± 0.31	1.10 ± 0.31	1.32 ± 0.77	0.08	0.66	0.99
	**Median** **(Q**_**25**_**, Q**_**75**_**)**	1.00 (1.00, 1.00)	1.00 (1.00, 1.00)	1.00 (1.00, 1.00)	1.00 (1.00, 1.00)			
**Adventitial inflammation score**	**Mean ± SD**	0.30 ± 0.70	0.20 ± 0.48	0.30 ± 0.65	0.39 ± 0.74	0.29	0.90	0.51
	**Median** **(Q**_**25**_**, Q**_**75**_**)**	0.00 (0.00, 0.00)	0.00 (0.00, 0.00)	0.00 (0.00,0.00)	0.00 (0.00, 0.25)			
**Inflammation severity score**	**Mean ± SD**	1.20 ± 0.66	1.03 ± 0.18	1.10 ± 0.31	1.18 ± 0.55	0.08	0.72	0.73
	**Median** **(Q**_**25**_**, Q**_**75**_**)**	1.00 (1.00, 1.00)	1.00 (1.00, 1.00)	1.00 (1.00, 1.00)	1.00 (1.00, 1.00)			
**% of struts with giant cells**	**Mean ± SD**	5.56 ± 14.55	7.54 ± 14.69	4.34 ± 9.94	9.37 ± 12.57	0.48	0.77	0.25
	**Median** **(Q**_**25**_**, Q**_**75**_**)**	0.00 (0.00, 5.77)	0.00 (0.00, 10.83)	0.00 (0.00, 0.00)	0.00 (0.00, 17.50)			
**Red blood cell extravasation score**	**Mean ± SD**	0.90 ± 0.31	0.93 ± 0.25	0.67 ± 0.48	0.96 ± 0.19	0.65	0.06	0.32
	**Median (Q**_**25**_**, Q**_**75**_**)**	1.00 (1.00, 1.00)	1.00 (1.00, 1.00)	1.00 (0.00, 1.00)	1.00 (1.00, 1.00)			
**% of struts with hemorrhage**	**Mean ± SD**	52.36 ± 28.10	50.60 ± 30.91	11.56 ± 18.43	58.76 ± 27.28	0.78	0.002	0.25
	**Median (Q**_**25**_**, Q**_**75**_**)**	55.56 (34.09, 69.81)	47.22 (27.08, 76.19)	0.00 (0.00, 19.17)	61.25 (48.21, 75.48)			

**Fig. 5:**
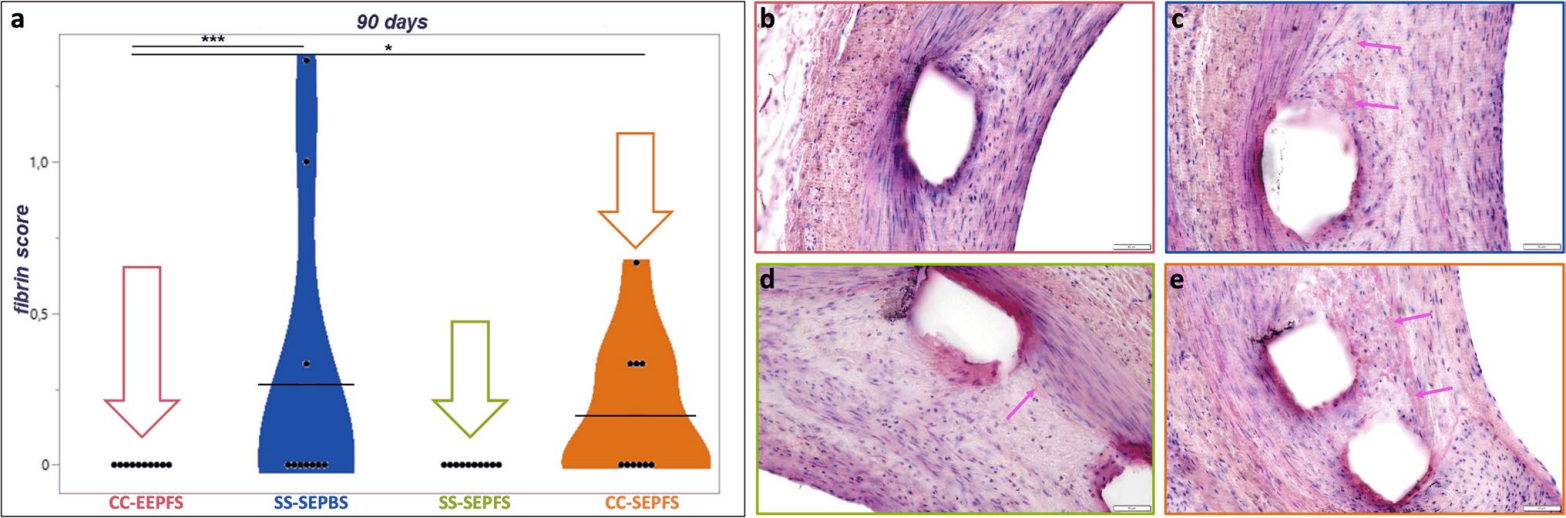
Histopathological analysis of fibrin score at 90 days follow-up. Fibrin score is visualized as a violin plot with the black bars indicating the mean value for each stent type. CC-EEPFS showed the greatest reduction of fibrin from 28 to 90 days follow-up and has a significantly lower fibrin score compared to SS-SEPBS (*p* = 0.001) and CC-SEPFS (*p* = 0.02) (**a**). Representative high power images show no or only traces of fibrin (pink arrows) in CC-EEPFS (**b**) and SS-SEPFS (**d**), while SS-SEPBS (**c**) and CC-SEPFS (**e**) still exhibit mild fibrin deposition adjacent to the stent struts. All images were taken at 20 × magnification. Scale bars depict 50 µm

At 90 days, the fibrin score differed significantly between CC-EEPFS, SS-SEPBS, and SS-SEPFS (see Fig. 5). CC-EEPFS showed a significantly lower fibrin score (mean = 0.00 ± 0.00) compared to SS-SEPBS (mean = 0.27 ± 0.52, *p* < 0.0001) and SS-SEPFS (mean = 0.17 ± 0.38, *p* = 0.02). Overall percentage of struts with fibrin was also lowest in CC-EEPFS (mean = 17.40 ± 14.03) compared to SS-SEPBS (mean = 22.88 ± 18.13, *p* < 0.0001] (see Table 4). Percentage of struts with surrounding hemorrhage were significantly lower in SS-SEPFS compared to CC-EEPFS (mean = 11.56 ± 18.43 vs. 52.36 ± 28.10, *p* = 0.002).

**Table 4:** Histologic comparison of vessel injury, healing and inflammatory response in stented cross sections at 90 days follow-up. Analysis includes mean ± SD, median and IQR of all stented cross sections

		CC-EEPFS (*n* = 10)	SS-SEPBS (*n* = 10)	SS-SEPFS (*n* = 10)	CC-SEPFS (*n* = 10)	*p* value CC-EEPFS vs. SS-SEPBS	*p* value CC-EEPFS vs. SS-SEPFS	*p* value CC-EEPFS vs.CC-SEPFS
**Injury Score**	**Mean ± SD**	1.17 ± 0.44	1.30 ± 0.47	1.24 ± 0.46	1.24 ± 0.46	0.005	0.98	0.17
	**Median (Q**_**25**_ **, Q**_**75**_ **)**	1.00 (1.00, 1.00)	1.00 (1.00, 2.00)	1.00 (1.00, 1.00)	1.00 (1.00, 1.00)			
**Injury Score circumferential Extension**	**Mean ± SD**	1.20 ± 0.41	1.37 ± 0.49	1.31 ± 0.54	1.31 ± 0.54	0.07	0.69	0.32
	**Median** **(Q**_**25**_ **, Q**_**75**_ **)**	1.00 (1.00, 1.00)	1.00 (1.00, 2.00)	1.00 (1.00, 1.50)	1.00 (1.00, 2.00)			
**Fibrin Score**	**Mean ± SD**	0.00 ± 0.00	0.27 ± 0.52	0.00 ± 0.00	0.17 ± 0.38	0.001	-	0.02
	**Median (Q**_**25**_ **, Q**_**75**_ **)**	0.00 (0.00, 0.00)	0.00 (0.00, 0.00)	0.00 (0.00, 0.00)	0.00 (0.00, 0.00)			
**% of Struts with Fibrin**	**Mean ± SD**	17.40 ± 14.03	45.42 ± 24.49	7.04 ± 9.24	22.88 ± 18.13	< 0.0001	0.02	0.20
	**Median** **(Q**_**25**_ **, Q**_**75**_ **)**	14.84 (8.52, 25.00)	42.26 (25.57, 64.29)	0.00 (0.00, 8.71)	22.22 (10.00, 35.71)			
**Inflammation score**	**Mean ± SD**	1.10 ± 0.31	1.20 ± 0.55	1.15 ± 0.36	1.00 ± 0.00	0.25	0.67	0.06
	**Median (Q**_**25**_ **, Q**_**75**_ **)**	1.00 (1.00, 1.00)	1.00 (1.00, 1.00)	1.00 (1.00, 1.00)	1.00 (1.00, 1.00)			
**Adventitial inflammation score**	**Mean ± SD**	0.20 ± 0.48	0.07 ± 0.25	0.19 ± 0.48	0.14 ± 0.35	0.13	0.99	0.50
	**Median (Q**_**25**_ **, Q**_**75**_ **)**	0.00 (0.00, 0.00)	0.00 (0.00, 0.00)	0.00 (0.00, 0.00)	0.00 (0.00, 0.00)			
**Inflammation severity score**	**Mean ± SD**	1.03 ± 0.18	1.07 ± 0.25	1.44 ± 1.93	1.00 ± 0.00	0.46	0.27	0.33
	**Median (Q**_**25**_ **, Q**_**75**_ **)**	1.00 (1.00, 1.00)	1.00 (1.00, 1.00)	1.00 (1.00, 1.00)	1.00 (1.00, 1.00)			
**% of struts with giant cells**	**Mean ± SD**	2.15 ± 5.55	4.91 ± 15.12	3.09 ± 11.36	0.00 ± 0.00	0.23	0.68	0.04
	**Median (Q**_**25**_ **, Q**_**75**_ **)**	0.00 (0.00, 0.00)	0.00 (0.00, 0.00)	0.00 (0.00, 0.00)	0.00 (0.00, 0.00)			
**Red blood cell extravasation score**	**Mean ± SD**	0.70 ± 0.47	0.80 ± 0.41	0.59 ± 0.50	0.79 ± 0.41	0.37	0.67	0.42
	**Median (Q**_**25**_ **, Q**_**75**_ **)**	1.00 (0.00, 1.00)	1.00 (1.00, 1.00)	1.00 (0.00, 1.00)	1.00 (1.00, 1.00)			
**% of struts with hemorrhage**	**Mean ± SD**	23.89 ± 23.20	30.40 ± 27.15	16.67 ± 19.59	24.93 ± 19.52	0.20	0.58	0.86
	**Median (Q**_**25**_ **, Q**_**75**_ **)**	17.42 (0.00, 47.50)	27.27 (2.78, 48.86)	9.09 (0.00, 33.33)	25.00 (10.00, 33.33)			

### SEM

**Fig. 6:**
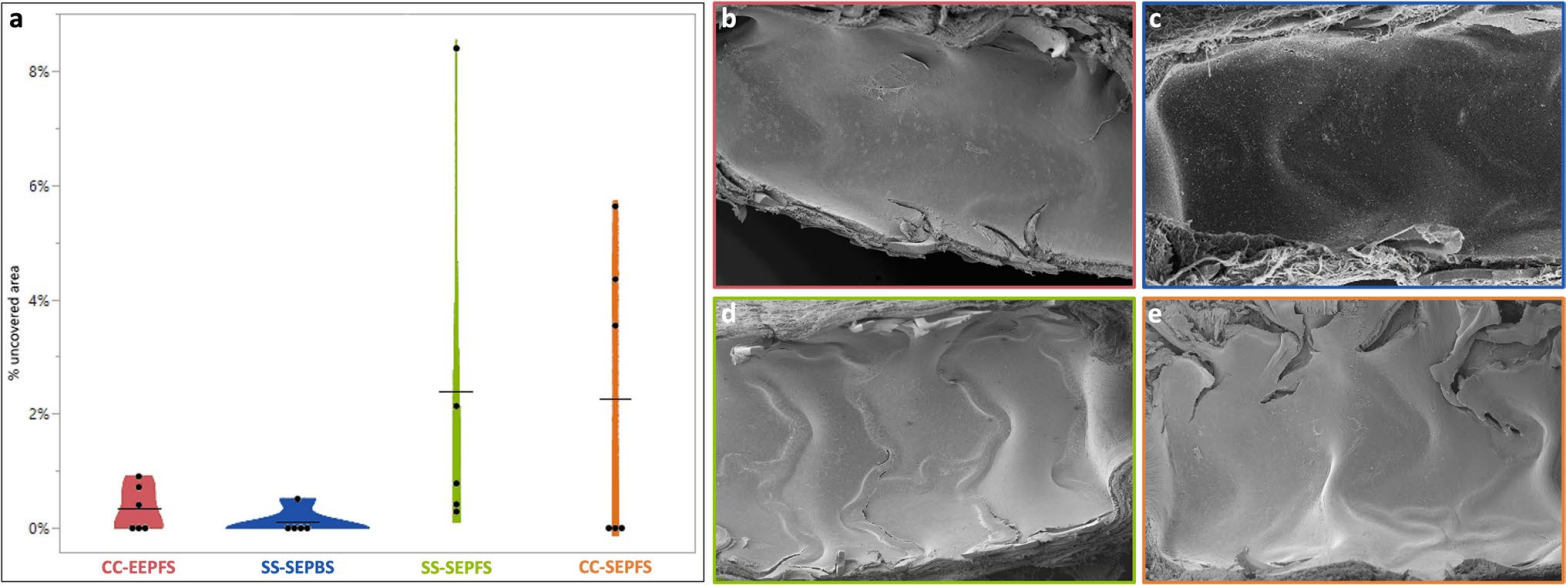
Percentage uncovered area per stent half at 14 days follow-up. Percentage uncovered area was measured on SEM images of stent halves and is visualized as a violin plot. The horizontal bars represent the mean values (CC-EEPFS *n* = 6, SS-SEPBS *n *= 5, SS-SEPFS *n* = 5, CC-SEPFS *n* = 6) (**a**). Representative SEM images of CC-EEPFS (**b**), SS-SEPBS (**c**), SS-SEPFS (**d**) and CC-SEPFS (**e**) stent halves show almost complete endothelial coverage for all stent types. Images were taken at 50 × magnification

Endothelialization was nearly complete in all devices at 14 days follow-up. There were no significant differences between CC-EEPFS and the control groups (see Fig. 6).

## Discussion

The current study aimed to investigate a novel hybrid DES technology avoiding polymer coating in juvenile porcine coronary arteries with regards to vascular healing and endothelialization, and to examine the relative contribution of the three different layer components of this novel DES platform, i.e. metallic backbone, drug carrier matrix and released substance by comparing to 3 different control groups. In this regard, the most salient findings of the study can be summarized as follows:

(i) The polymer-free CC-EEPFS showed improved neointimal strut coverage compared to biodegradable polymer-coated SS-SEPBS at 28 days and significantly lower overall neointimal growth compared to biodegradable polymer-coated SS-SEPBS at 90 days.
(ii) Fibrin deposition was highest in the polymer-free CC-EEPFS at 28 days and resolved to reach significantly lower fibrin score and percentage of struts with fibrin relative to polymer-coated SS-SEPBS after 90 days
(iii) Fibrin deposition (struts with fibrin) was significantly lower in the polymer-free CC-EEPFS compared to polymer-free SS-SEPFS at 90 days.
(iv) Scanning electron microscopy revealed near complete endothelialization in all study groups after 14 days.

### Comparative Vascular Healing and Endothelialization among Study Groups

The current study showed that novel polymer-free hybrid DES releasing everolimus resulted in improved strut coverage compared to biodegradable polymer-coated DES at 28 days, in combination with reduced neointimal growth in juvenile porcine coronary arteries after 90 days. The likely explanation for this favorable vascular healing profile is the achievement of high drug concentrations in the surrounding tissue in the first 28 days following implantation, which was mirrored by increased fibrin deposition in the vicinity of stent struts of CC-EEPFS relative to control devices. We have previously reported that fibrin deposition reflects indirect drug effect in preclinical studies investigating DES [3] and that the presence of fibrin surrounding stent struts can be considered effective drug release in the early stage of vascular healing. Importantly, fibrin deposition resolved to low levels compared to control devices after 90 days in this animal model, which supports the notion of improved vascular healing in this novel hybrid DES. Since neointimal growth was lower in CC-EEPFS relative to its biodegradable polymer-coated control device after 90 days, it is likely that improved vascular healing in the early phase (28 days) resulted in diminished neointimal growth in the intermediate phase (90 days). One of the landmark findings in seminal pathology studies of first-generation DES was the prevalence of delayed vascular healing, characterized by prolonged fibrin deposition, delayed endothelialization and sustained inflammation relative to bare metal stent counterparts [3]. Resolving fibrin deposition marked improved vascular healing in second-generation DES studies at autopsy, which went along with improved strut coverage and more balanced neointimal growth [10]. Consequently, innovative stent designs should focus on achieving these cornerstones of vascular healing, to ultimately help reduce accrual of late vascular events, likely related to delayed vascular healing. In a prior study comparing different DES technologies with regards to their potential to delay re-endothelialization following implantation, we reported that 14 days may be the optimal time point to investigate differences in re-endothelialization in juvenile animal models [11]. Against this background, we have shown that CC-EEPFS resulted in a similar pace of re-endothelialization relative to commercially available biodegradable polymer-coated DES after 14 days, despite the use of probucol as drug carrier matrix. This is the first preclinical study to demonstrate that combined release of *limus* analogs and probucol resulted in superior anti-restenotic drug effect after 90 days in porcine coronary arteries, while preserving rapid re-endothelialization and strut coverage. These data support the notion that relevant anti-proliferative drug-effect and balanced vascular healing can be achieved with a hybrid coating strategy, avoiding potential adverse biological reactions arising from polymer coatings. In addition, SS-SEPFS was shown to have lower percentage of struts with surrounding hemorrhage, which may be explained by the lower tissue slicing capacity of stainless-steel struts at larger dimensions compared to those of CC-EEPFS, which was made from Cobalt-Chrome.

### Investigation of Different Stent Components in Preclinical Studies

The strength of preclinical studies in the field of DES technology must be seen in their potential to examine individual stent components and their impact towards vascular healing. In this regard, the current study addressed this topic by using 3 different control devices, each of them enabling to control for individual components of CC-EEPFS, and in this way facilitating improved understanding of specific vascular reactions. Against this background, we found that the polymer-free CC-EEPFS showed improved overall vascular healing relative to the polymer-coated control (SS-SEPBS) DES. Furthermore, the 2 additional control devices (SS-SEPFS and CC-SEPFS), which shared the absence of a polymer coating while applying a probucol carrier matrix to release sirolimus, also trended towards reduced neointimal growth and fibrin deposition after 90 days compared to polymer-coated SS-SEPBS. This finding suggests that not only can sufficient drug effect be achieved in the absence of pro-inflammatory polymer coatings, but it may also improve vascular healing in the intermediate stage (90 days), resulting in diminished neointimal growth. Another salient finding of this study was that CC-EEPFS showed reduced fibrin deposition relative to SS-SEPFS, which consisted of a stainless-steel backbone, releasing sirolimus from the same probucol carrier matrix as compared to CC-EEPFS. An important distinguishing feature was the slightly higher strut thickness in this group, which likely resulted in greater vascular injury and, consequently, fibrin deposition. This finding is in strong support of prior preclinical and clinical studies highlighting the relevance of strut thickness towards vascular healing and angiographic late lumen loss [12], while the metallic backbone material may be of lower relevance in this respect. Furthermore, the relative similarity in vascular healing and neointimal growth among CC-EEPFS and CC-SEPFS, which only differed in the drug (everolimus in CC-EEPFS vs. sirolimus in CC-SEPFS) applied to the probucol carrier matrix, suggested that the switch from sirolimus to everolimus may not result in relevant deviation with regards to biological responses amongst these devices. Along these lines, we have previously reported about the lack of significant differences in vascular healing and re-endothelialization when three different *limus* analogs (everolimus, zotarolimus and sirolimus) were released from an identical stent platform with similar release kinetics in a preclinical rabbit study [13].

## Clinical Relevance

This preclinical study offers pathophysiological insight into the performance of a novel polymer-free hybrid DES. Existing polymer-coated DES are limited by accrual of late adverse clinical events likely resulting from long-term inflammatory responses to the polymer. By demonstrating early endothelialization and attenuated neointimal growth in a porcine coronary model, the results highlight favorable biological responses to a polymer-free, probucol-based matrix. These findings support the potential of this stent platform to overcome limitations associated with polymer-coated devices and provide a strong foundation for future clinical evaluation.

## Limitations

The current study utilized juvenile healthy pigs to investigate vascular healing among different DES designs. Consequently, the absence of underlying atherosclerotic disease conditions may prevent from drawing definite conclusions relevant for the performance of these devices in humans. Yet, preclinical studies represent an important means to understand biocompatibility and safety of novel devices prior to first-in-human use.

## Conclusions

The current study provided evidence for delayed but effective drug release from a novel hybrid technology DES releasing everolimus from a probucol carrier matrix, in the absence of (biodegradable) polymer coating. Favorable vascular healing between 28 and 90 days suggests improved performance relative to benchmark devices, which needs confirmation in dedicated clinical trials.

## Supplementary Information

Below is the link to the electronic supplementary material.

Supplementary file1 (DOCX 25 KB)

## Data Availability

Data will be made available on request. Figures and graphical abstract were generated with BioRender and can be viewed/cited under the following URL: https://BioRender.com/g28p691.

## Abbreviations

BMS: Bare metal stent
CC-EEPFS: Cobalt chromium everolimus eluting polymer free stent
CC-SEPFS: Cobalt chromium sirolimus eluting polymer free stent
CoCr: Cobalt chrome
DES: Drug-eluting stent
HE: Hematoxylin and eosin
i.c.: Intracoronary
IEL: Internal elastic lamina
i.m.: Intramuscular
IQR: Interquartile range
i.v.: Intravenous
LAD: Left anterior descending
LCx: Left circumflex
MMA: Methyl methacrylate
MV: Medium vessel
PCI: Percutaneous coronary intervention
PLA: Polylactic acid
QCA: Quantitative coronary angiography
RCA: Right coronary artery
SD: Standard deviation
SEM: Scanning electron microscopy
SS: Stainless steel
SS-SEPBS: Stainless steel sirolimus eluting polymer based stent
SS-SEPFS: Stainless steel sirolimus eluting polymer free stent
SV: Small vessel
VVG: Verhoeff van Gieson

## Declarations

No human studies were carried out by the authors for this article.

All institutional and national guidelines for the care and use of laboratory animals were followed and approved by the appropriate institutional committees.
