# Supplementary material for: Preclinical Assessment of a Novel Polymer-Free Hybrid Drug Eluting Stent
Source: J Cardiovasc Transl Res. 2025 Aug 15;18(5):1383–94. doi: 10.1007/s12265-025-10680-5 (PMC12630216; doi:10.1007/s12265-025-10680-5)
Supplement: Supplementary file 1 — Supplementary file1 (DOCX 25 KB) [file 12265_2025_10680_MOESM1_ESM.docx]

**Supplementary table 1:** Summary of the different characteristics of each device.

| **Study device** | **Backbone** | **Matrix** | **Drug** | **Drug Load** | **Strut Thickness** | **Strut Width** | **Markers** |
| --- | --- | --- | --- | --- | --- | --- | --- |
| **CC-EEPFS** | ^1^CoCr | Probucol | Everolimus | 2.6 µg/mm^2^ or 12.5 µg per mm of stent length | 68 µm | 78 µm | 2 Nos Platinum Iridium Markers in the Balloon |
|  |  |  |  |  | 79 µm | 88 µm |  |
| **SS-SEPBS** | ^2^SS | ^3^PLA | Sirolimus |  | 87 µm | 96 µm |  |
|  |  |  |  |  | 96 µm | 120 µm |  |
| **SS-SEPFS** | SS | Probucol | Sirolimus |  | 87 µm | 96 µm |  |
|  |  |  |  |  | 96 µm | 120 µm |  |
| **CC-SEPFS** | CoCr | Probucol | Sirolimus |  | 68 µm | 78 µm |  |
|  |  |  |  |  | 79 µm | 88 µm |  |

^1^CoCr – Cobalt Chromium, ^2^SS – Stainless Steel, ^3^PLA – Poly-lactic acid

**Supplementary table 2:** Number of implanted stents per stent size. Numbers in round brackets depict total amount of implanted devices, numbers without brackets the amount of stents analyzed.

| **Study device** | **Stent size** | **Timepoint of sacrifice** | **Nr. of stents analyzed** |
| --- | --- | --- | --- |
| CC-EEPFS | 2.75x12 | 14 d | 0 |
|  |  | 28 d | 1 |
|  |  | 90 d | 1 |
|  | 3.0x12 | 14 d | 1 |
|  |  | 28 d | 3 |
|  |  | 90 d | 4 |
|  | 3.5x12 | 14 d | 0 |
|  |  | 28 d | 0 |
|  |  | 90 d | 0 |
|  | 3.5x16 | 14 d | 1 |
|  |  | 28 d | 4 |
|  |  | 90 d | 4 (5) |
|  | 3.5x18 | 14 d | 1 |
|  |  | 28 d | 0 |
|  |  | 90 d | 0 |
|  | 4.0x12 | 14 d | 0 |
|  |  | 28 d | 2 |
|  |  | 90 d | 1 |
| SS-SEPBS | 2.75x12 | 14 d | 0 |
|  |  | 28 d | 1 |
|  |  | 90 d | 1 |
|  | 3.0x12 | 14 d | 1 |
|  |  | 28 d | 4 |
|  |  | 90 d | 3 (4) |
|  | 3.5x12 | 14 d | 2 |
|  |  | 28 d | 0 |
|  |  | 90 d | 0 |
|  | 3.5x16 | 14 d | 0 |
|  |  | 28 d | 5 |
|  |  | 90 d | 5 |
|  | 3.5x18 | 14 d | 0 |
|  |  | 28 d | 0 |
|  |  | 90 d | 0 |
|  | 4.0x12 | 14 d | 0 |
|  |  | 28 d | 0 |
|  |  | 90 d | 1 |
| SS-SEPFS | 2.75x12 | 14 d | 0 |
|  |  | 28 d | 4 |
|  |  | 90 d | 1 |
|  | 3.0x12 | 14 d | 0 |
|  |  | 28 d | 3 |
|  |  | 90 d | 7 |
|  | 3.5x12 | 14 d | 0 |
|  |  | 28 d | 0 |
|  |  | 90 d | 0 |
|  | 3.5x16 | 14 d | 2 |
|  |  | 28 d | 2 |
|  |  | 90 d | 0 |
|  | 3.5x18 | 14 d | 0 |
|  |  | 28 d | 0 |
|  |  | 90 d | 0 |
|  | 4.0x12 | 14 d | 1 |
|  |  | 28 d | 1 |
|  |  | 90 d | 2 |
| CC-SEPFS | 2.75x12 | 14 d | 1 |
|  |  | 28 d | 3 |
|  |  | 90 d | 0 |
|  | 3.0x12 | 14 d | 1 |
|  |  | 28 d | 1 |
|  |  | 90 d | 3 |
|  | 3.5x12 | 14 d | 0 |
|  |  | 28 d | 0 |
|  |  | 90 d | 0 |
|  | 3.5x16 | 14 d | 0 |
|  |  | 28 d | 4 |
|  |  | 90 d | 5 (6) |
|  | 3.5x18 | 14 d | 1 |
|  |  | 28 d | 2 |
|  |  | 90 d | 0 |
|  | 4.0x12 | 14 d | 0 |
|  |  | 28 d | 0 |
|  |  | 90 d | 2 |

**Supplementary table 3:** Description of semi-quantitative histology scores.

| **Attribute** | **Score** | **Description of Assigned Weight** |
| --- | --- | --- |
| **Injury Score** | **0** | Internal elastic lamina (IEL) intact, endothelium typically denuded, media may be compressed but not lacerated |
|  | **1** | IEL lacerated, media typically compressed but not lacerated |
|  | **2** | IEL lacerated, media visibly lacerated, external elastic lamina (EEL) intact but may be compressed |
|  | **3** | EEL lacerated, typically large lacerations of media extending through EEL, covered stent struts sometimes residing in adventitia |
| **Inflammation Score** | **0** | <25% struts with fewer than 10 inflammatory cells |
|  | **1** | Up to 25% struts with greater than 10 inflammatory cells |
|  | **2** | 25-50% struts with greater than 10 inflammatory cells |
|  | **3** | >50% struts with greater than 10 inflammatory cells |
|  | **4** | 2 or more struts associated granulomatous inflammatory reactions |
| **Fibrin Score** | **0** | None to focal residual fibrin involving any portion of the vessel |
|  | **1** | Mild fibrin deposition involving <25% of the covered stentstruts |
|  | **2** | Moderate fibrin deposition involving 25% to 50% of the covered stentstruts |
|  | **3** | Heavy deposition of fibrin surrounding >50% of the covered stentstruts |
| **Adventitial Inflammation Score** | **0** | No inflammation to minimal interspersed inflammatory cells anywhere in the adventitia |
|  | **1** | Mild peripheral inflammatory infiltration or focally moderated in <25% of adventitial area |
|  | **2** | Moderate peripheral inflammatory infiltration or focally marked in 25-50% of adventitial area |
|  | **3** | Heavy peripheral inflammatory infiltration or focally marked in >50% of adventitial area |

**Supplementary table 4:** Description of semi-quantitative histology scores for circumferential extension.

| **Attribute** | **Score** | **Description of Assigned Weight** |
| --- | --- | --- |
| **Injury Score**  (circumferential) | **0** | No injury |
|  | **1** | < 25% of the vessel circumference showing disruption of the EEL, IEL and media respectively |
|  | **2** | 25-50% of the vessel circumference showing disruption of the EEL, IEL and media respectively |
|  | **3** | >50% of the vessel circumference showing disruption of the EEL, IEL and media respectively |
| **Giant Cell Score** (circumferential) | **0** | None to focal giant cells involving any portion of the vessel |
|  | **1** | Mild giant cell infiltration involving <10% of the circumference of the vessel |
|  | **2** | Moderate giant cell infiltration involving 10% to 25% of the circumference of the vessel |
|  | **3** | Heavy giant cell infiltration involving >25% of the circumference of the vessel |
| **Neointimal Fibrin Deposition**  score to be used for neointimal fibrin between covered stentand lumen and between the covering material layers | **0** | none to focal interstitial fibrin minimal spotting of fibrin generally consistent with background levels |
|  | **1** | < 10% of circumference showing interstitial fibrin |
|  | **2** | 10-25% of circumference showing interstitial fibrin |
|  | **3** | > 25% of the circumference showing interstitial fibrin |
| **Red Blood Cell Extravasation Score**  supplemental to percent of associated struts | **0** | No red blood cell extravasation |
|  | **1** | < 25% red blood cell extravasation |
|  | **2** | 25-50% red blood cell extravasation showing disruption of the EEL, IEL and media respectively |
|  | **3** | >50% red blood cell extravasation |

**Supplementary table 5:** Description of inflammation severity scoring.

| **Severity*** | | | **Circumferential Extension** | | | |
| --- | --- | --- | --- | --- | --- | --- |
|  |  |  | **<25%** | **>25-50%** | **>50-70%** | **>75%** |
| **0** | | | 0 | 0 | 0 | 0 |
| **1** | | | 1 | 1 | 1 | 1 |
| **2** | | | 1 | 1 | 2 | 2 |
| **3** | | | 2 | 2 | 3 | 4 |
| **4** | | | 2 | 3 | 4 | 4 |
| *Severity scale is defined as: | | | | | | |
|  | **0** | No inflammation present; | | | | |
|  | **1** | Rare inflammatory cells present; | | | | |
|  | **2** | Mild infiltrate that does not efface and is not the predominant component of the associated tissue; | | | | |
|  | **3** | Infiltrate that largely effaces or predominates other tissue components; and | | | | |
|  | **4** | Infiltrate that effaces other tissue components. | | | | |
|  | | | | | | |

**Supplementary table 6:** QCA comparison for each stent at 28 days follow-up.

|  | | **CC-EEPFS**  **(n=10)** | **SS-SEPBS**  **(n=10)** | **SS-SEPFS**  **(n=10))** | **CC-SEPFS**  **(n=10)** | **p value**  **CC-EEPFS vs.  SS-SEPBS** | **p value**  **CC-EEPFS vs.  SS-SEPFS** | **p value**  **CC-EEPFS vs. CC-SEPFS** |
| --- | --- | --- | --- | --- | --- | --- | --- | --- |
| **Minimum diameter, mm** | **Mean ± SD** | 2.09 ± 0.42 | 2.25 ± 0.19 | 1.97 ± 0.60 | 1.97 ± 0.60 | 0.21 | 0.82 | 0.60 |
|  | **Median (Q_25_, Q_75_)** | 1.94 (1.81, 2.37) | 2.23 (2.15, 2.32) | 2.18 (1.53, 2.46) | 2.18 (1.53, 2.46) |  |  |  |
| **Mean diameter, mm** | **Mean ± SD** | 2.30 ± 0.37 | 2.52 ± 0.26 | 2.25 ± 0.52 | 2.25 ± 0.52 | 0.08 | 0.54 | 0.81 |
|  | **Median (Q_25_, Q_75_)** | 2.10 (2.06, 2.53) | 2.54 (2.30, 2.75) | 2.45 (1.78, 2.66) | 2.45 (1.78, 2.66) |  |  |  |
| **Diameter stenosis, %** | **Mean ± SD** | 7.14 ± 3.87 | 8.64 ± 3.16 | 10.17 ± 5.34 | 10.17 ± 5.34 | 0.28 | 0.22 | 0.18 |
|  | **Median (Q_25_, Q_75_)** | 6.94 (4.99, 7.52) | 9.04 (6.37, 11.62) | 8.00 (6.99, 13.88) | 8.00 (6.99, 13.88) |  |  |  |
| **Late lumen loss, mm** | **Mean ± SD** | 0.38 ± 0.36 | 0.29 ± 0.20 | 0.63 ± 0.29 | 0.63 ± 0.29 | 0.35 | 0.50 | 0.12 |
|  | **Median (Q_25_, Q_75_)** | 0.38 (0.24, 0.47) | 0.23 (0.14, 0.37) | 0.55 (0.45, 0.74) | 0.55 (0.45, 0.74) |  |  |  |

**Supplementary table 7:** QCA comparison for each stent at 90 days follow-up.

|  | | **CC-EEPFS**  **(n=10)** | **SS-SEPBS**  **(n=10)** | **SS-SEPFS**  **(n=10))** | **CC-SEPFS**  **(n=10)** | **p value**  **CC-EEPFS vs.  SS-SEPBS** | **p value**  **CC-EEPFS vs.  SS-SEPFS** | **p value**  **CC-EEPFS vs. CC-SEPFS** |
| --- | --- | --- | --- | --- | --- | --- | --- | --- |
| **Min. diameter, mm** | **Mean ± SD** | 2.29 ± 0.44 | 1.97 ± 0.47 | 2.25 ± 0.46 | 2.25 ± 0.46 | 0.14 | 0.48 | 0.84 |
|  | **Median (Q_25_, Q_75_)** | 2.30 (1.98, 2.64) | 1.92 (1.81, 2.15) | 2.31 (1.97, 2.66) | 2.31 (1.97, 2.66) |  |  |  |
| **Mean diameter, mm** | **Mean ± SD** | 2.49 ± 0.42 | 2.21 ± 0.42 | 2.43 ± 0.42 | 2.43 ± 0.42 | 0.17 | 0.80 | 0.76 |
|  | **Median (Q_25_, Q_75_)** | 2.48 (2.27, 2.87) | 2.19 (2.10, 2.38) | 2.46 (2.20, 2.82) | 2.46 (2.20, 2.82) |  |  |  |
| **Diameter stenosis, %** | **Mean ± SD** | 7.18 ± 3.21 | 9.31 ± 4.09 | 6.27 ± 2.74 | 6.27 ± 2.74 | 0.20 | 0.10 | 0.51 |
|  | **Median (Q_25_, Q_75_)** | 6.01 (5.36, 8.70) | 8.13 (6.31, 11.10) | 5.59 (4.93, 8.14) | 5.59 (4.93, 8.14) |  |  |  |
| **Late lumen loss, mm** | **Mean ± SD** | 0.26 ± 0.22 | 0.50 ± 0.28 | 0.36 ± 0.26 | 0.36 ± 0.26 | 0.06 | 0.86 | 0.35 |
|  | **Median (Q_25_, Q_75_)** | 0.23 (0.15, 0.42) | 0.51 (0.36, 0.68) | 0.30 (0.21, 0.60) | 0.30 (0.21, 0.60) |  |  |  |
